# The Intestinal Barrier—Shielding the Body from Nano- and Microparticles in Our Diet

**DOI:** 10.3390/metabo12030223

**Published:** 2022-03-02

**Authors:** Marlene Schwarzfischer, Gerhard Rogler

**Affiliations:** Department of Gastroenterology & Hepatology, University Hospital Zurich, 8091 Zurich, Switzerland; marlene.schwarzfischer@usz.ch

**Keywords:** epithelial cells, intestinal mucosa, nanoparticles, titanium dioxide, inflammasome, microplastic, nanoplastic

## Abstract

Nano- and microparticles are an implicit part of the human diet. They are unknowingly ingested with our food that contains them as additives or pollutants. However, their impact on human health is not yet understood and controversially discussed. The intestinal epithelial barrier shields our body against exogenous influences, such as commensal bacteria, pathogens, and body-foreign particles and, therefore, protects our body integrity. Breakdown of the intestinal epithelial barrier and aberrant immune responses are key events in the pathogenesis of inflammatory bowel disease (IBD). Epithelial lesions might enable systemic translocation of nano- and microparticles into the system, eventually triggering an excessive immune response. Thus, IBD patients could be particularly vulnerable to adverse health effects caused by the ingestion of synthetic particles with food. The food-additive titanium dioxide (TiO_2_) serves as a coloring agent in food products and is omnipresent in the Western diet. TiO_2_ nanoparticles exacerbate intestinal inflammation by activation of innate and adaptive immune response. Because of serious safety concerns, the use of TiO_2_ as a food additive was recently banned from food production within the European Union. Due to environmental pollution, plastic has entered the human food chain, and plastic microparticles have been evidenced in the drinking water and comestible goods. The impact of plastic ingestion and its resulting consequences on human health is currently the subject of intense research. Focusing on TiO_2_ and plastic particles in the human diet and their impact on epithelial integrity, gut homeostasis, and intestinal inflammation, this review is addressing contemporary hot topics which are currently attracting a lot of public attention.

## 1. Introduction

The intestinal epithelial is critical for absorbing nutrients and shielding the body from harmful exogenous factors. It is composed of a single layer of intestinal epithelial cells (IEC) sealed by multi-protein complexes called tight junctions, which control the passage of water, ion, and solutes through the paracellular way [1,2,3]. IECs restrict the entrance of commensal bacteria by secretion of mucin and anti-microbial molecules while tight junctions prevent the entry of pathogens and regulate electrolyte secretion. Furthermore, they selectively allow for the access of antigens derived from food or commensal bacteria to induce oral tolerance [4,5]. Therefore, it is evident that disruption of the epithelial barrier may lead to mucosal inflammation [6].

**Figure 1:**
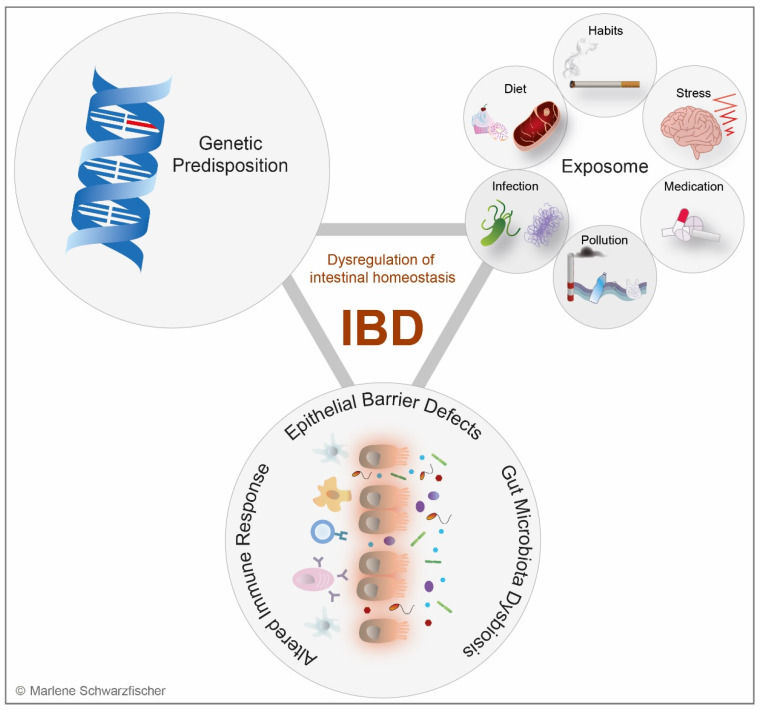
IBD—a multifactorial disease. IBD pathogenesis is driven by an interplay of genetic and environmental risk factors, causing epithelial barrier defects, microbial dysbiosis, and excessive up- or downregulation of the immune response, which culminates in dysregulation of the intestinal homeostasis and intestinal inflammation. Environmental factors can influence the onset and course of IBD and are collectively referred to as the exposome. For example, cigarette smoking, stress, medication, air and water pollution, infections, and dietary preferences were shown to modulate IBD risk.

Disturbances of the mucosal epithelial barrier contribute to the pathogenesis of inflammatory bowel diseases (IBD) and other systemic conditions. IBD is a term for a group of chronic disorders of the gastrointestinal tract (GIT) which are characterized by relapsing inflammation [7]. Considering the manifestation of the inflammation, IBD can be sub-divided into two primary conditions: Crohn’s disease (CD) may affect any part of the digestive system from the mouth to the anus [7], whereas ulcerative colitis (UC) is restricted to the large bowel, with pronounced inflammation in the rectum [7]. Triggered by genetic and environmental risk factors, IBD has become a prototype for a multifactorial disease [7,8]. IBD onset is characterized by dysregulation of intestinal homeostasis, triggered by epithelial barrier defects, altered immune response, and dysbiosis of the gut microbiome [9] (Figure 1). This imbalance subsequently results in bacterial translocation across the intestinal barrier and provokes aberrant activation of inflammatory cascades [10]. A leaky intestinal barrier with increased intestinal permeability was first reported more than 20 years ago in patients with CD [11]. However, for decades, it was not clear whether it was a cause or result of the disease. A recent patient study supports the hypothesis that the breakdown of the epithelial barrier is a causative key event in CD pathogenesis and may serve as a biomarker for CD onset [12].

There is evidence that IECs are activated and that the epithelial barrier integrity is disturbed in IBD patients even before the onset of intestinal inflammation [13,14,15,16]. In CD, increased epithelial permeability might result from a decreased expression of the tight junction proteins occludin, claudin 5/8, and increased presence of the pore-forming protein claudin 2 [17]. In UC, apoptosis of IECs causes focal epithelial lesions, which are mainly responsible for the observed barrier dysfunction [17,18,19]. Furthermore, overexpression of pro-inflammatory cytokines, including tumor necrosis factor (TNF) and interferon (IFN) γ, modulate tight junctions, induce apoptosis in epithelial cells and, therefore, impair barrier integrity in IBD patients [20,21,22,23]. Increased permeability subsequently allows high-penetration of antigens and loss of essential ions and water, resulting in diarrhea [19,20]. Genetic variants in genes belonging to the Janus kinases (JAKs) and signal transducers and activators of transcription (STATs) have been associated with epithelial barrier defects and increased IBD risk. Barrier integrity is regulated by the JAK-STAT signaling pathway, controlling paracellular permeability and epithelial cell death. Suppression of these signaling cascades was proven to have a substantial therapeutic potential for IBD treatment [23]. Assessment of intestinal barrier integrity is tricky, as demonstrated recently by Power et al. refuting several studies that proclaimed barrier-associated molecules zonula occludens 1 (ZO-1) and intestinal fatty acid-binding proteins (I-FABP) to be sufficient serological markers for quantification of epithelial damage. Based on these findings, the authors raise the question of whether physiologic measurement of intestinal permeability necessarily correlates with changes in tight junction structures [24]. In turn, increased anti-microbial antibody responses have been associated with an elevated CD risk and may serve as an early pre-disease marker for CD onset [25].

## 2. Environmental Factors Contribute to Epithelial Activation and Barrier Defects

Environmental risk factors for IBD are numerous and almost inevitably present in our daily life, and their impact on IBD risk, onset, and the disease course can hardly be captured. Disease-relevant external factors are collectively referred to as the exposome. This index allows assessment of the total environmental (non-genetic) exposure individuals face during their lifetime and estimation of the resulting impact on their health [26]. In terms of IBD, the most relevant environmental factors associated with the disease are personal habits, psychological stress, medication, environmental pollution, infection, and diet [8,27] (Figure 1).

Progressing with industrialization, environmental pollution is increasing around the globe. Global plastic contamination is one of the biggest challenges of our times. The increasing abundance of NP and MP particles in the human diet raises concerns about their safety and potential impacts on gastrointestinal health. Previous studies reported that plastic ingestion might affect barrier integrity and induce histological changes, alterations of gut physiology, and gut microbiota dysbiosis [28,29,30,31,32]. Rising exposure to air pollutants such as sulfur dioxide (SiO_2_), nitric oxide (NO), and particulate matter was suspected to increase the risk of IBD onset [27,33,34,35]. In addition, air pollutants were hypothesized to alter the microbiome composition and therefore modify IBD risk. However, associations are complex and require further studies [36,37]. The impact of water pollutants on IBD risk is of great interest as antagonists of steroid receptors, endocrine-disrupting chemicals, phthalic acid, and nonylphenols, evidenced in bottled water, might interfere with therapy targeting the steroid hormone metabolism [8,27,38,39,40,41].

Our diet is an important exogenous factor that plays an undisputed role in IBD onset and progression [8,27,42,43]. Our dietary habits might impact IBD risk and disease outcome in several ways, directly modulating the gut microbiome, influencing the intestinal barrier’s integrity and permeability, and affecting the immune system [42]. Strikingly, dietary changes can alter the intestinal microbiome within 24 h [43]. Specific diets have been associated with a reduced or increased IBD risk [44].

Dietary management, aiming for a balanced diet, was said to impact the disease course positively and, therefore, improve the well-being of IBD patients [45,46,47,48]. For example, high dietary fiber intake was shown to modify the gut microbiome composition beneficially and to reduce intestinal inflammation [49]. Furthermore, a high fiber diet has been associated with a decreased CD risk [50]. In addition, flavonoids were found to possess anti-inflammatory capacities and suppress intestinal inflammation via modulation of the enteroendocrine system [51].

In contrast, food antigens might be an important trigger of intestinal inflammation as most IBD patients report intolerance to certain dietary constituents [52]. Western diet, rich in sugar, animal proteins, and polyunsaturated fatty acids (PUFAs), might predispose to IBD [53,54,55,56]. Several studies have shown that the Western diet promotes intestinal inflammation via modulation of barrier integrity and the gut microbiome, resulting in altered gut homeostasis [53,57,58,59,60,61,62,63]. A high-sugar diet affected the intestinal epithelial integrity in mice, increased their susceptibility to dextran sodium sulfate (DSS)-induced colitis, and altered their gut microbiome [64]. Similar findings were made when mice were fed a diet rich in animal protein, which resulted in pro-inflammatory macrophages’ response and exacerbation of DSS-induced colitis [55]. A high-fat diet was shown to induce oxidative stress in the murine mucosa, triggering mucosal inflammation and increasing barrier permeability [65]. Western diet is characterized by an imbalance of n-3 and n-6 PFUAs in favor of n-6 PFUAs [66,67]. Although epidemiologic studies identified n-3 PFUAs to have anti-inflammatory properties and prevent UC, high n-6 PFUAs consumption was found to significantly increase the risk for UC onset and other inflammatory diseases [66]. Considering the rising prevalence and incidence of IBD in westernized countries around Asia, North America, and the Middle East, diet westernization seems likely to be the significant driver of IBD around the globe [27,59,68,69,70].

Food products from the Western world are often processed and preserved using food additives to optimize flavor, color, and texture artificially. A recent review by Raoul et al. nicely describes the associations of food additives and IBD concerning alterations in the gut microbiome and impacts on gastrointestinal homeostasis [71]. Food-processing often involves the usage of nano-additives [72], including silver (Ag) (E174), iron oxide (FeO) (E172), silicon dioxide (SiO_2_) (E551), and titanium dioxide (TiO_2_) (E171) nanoparticles, which are nowadays an implicit part of the human diet. Ghebretatios et al. recently assessed the use of nanoparticles in the food industry and the impact of nanoparticle-induced microbiota changes in the pathogenesis of intestinal diseases [72]. Penetration of the intestinal epithelium by body-foreign particles is determined by their diameter [73,74]. It is assumed that, due to their smaller size, nanoparticles possess higher bioavailability which allows for increased translocation into the system. Body-foreign particles below 150 µm in diameter penetrate the intestinal epithelium, while micro- and nano-sized particles below 2.5 µm in diameter are absorbed by microfold cells (M-cells) in Payer’s patches [73]. Therefore, it needs to be investigated more closely whether the ingestion of nano- and microparticles with our diet may adversely affect human health, especially gastrointestinal homeostasis.

## 3. TiO_2_ in the Human Diet—A Constant Companion

Dietary preferences are tightly linked to the unintentional consumption of food additives, such as stabilizers, antioxidants, flavor enhancers, or food coloring agents. Due to its shiny, white, and bright appearance, TiO_2_ is a popular food-coloring agent and is highly abundant in the Western diet [75,76]. TiO_2_ nanoparticles find use in comestible goods, medicine, and personal care products to optimize their appearance and to meet customers’ preferences [76,77].

Following the Codex Alimentarius, published by a committee of experts delegated from the Food and Agriculture Organization (FAO) and the World Health Organization (WHO), forming the Joint FAO/WHO Expert Committee on Food Additives (JECFA), the use of food-grade TiO_2_ was approved by the United States Food and Drug Administration (USFDA) in 1966 as first [78,79] and three years later by the European Food Safety Authority (EFSA) [79,80]. EFSA permitted the use of TiO_2_ for milk and dairy products, cheese products, preparation of fruits and vegetables, chewing gums, confectionery, edible ices, decorations, coatings and fillings of pastry and fine bakery ware, breakfast cereals, processed nuts, noodles, batters, the casing of meat products, processing of fish and fishery products, sauces, salad toppings, soups and broths, protein and dietary foods, flavored drinks, alcoholic drinks, and other foodstuffs [80]. Popular non-food sources of oral TiO_2_ intake are toothpaste and pills [81]. By 2016, the global volume consumption of TiO_2_ nanoparticles exceeded 6 million metric tons, and consumption is projected to increase further [82], pushing the market to USD 14.12 billion by 2021 [83]. TiO_2_ mainly occurs in three structures: brookite, rutile, and anatase, whereby only rutile and anatase particles may be used in food [84]. The size of the particles found in food products varies between 10–350 nm, with a relevant fraction of particles below 100 nm [75,85,86,87,88]. According to a regulation from the European Commission in 2011, the use of TiO_2_ in food must be indicated in the list of ingredients, and the application of TiO_2_ nanoparticles must be explicitly labeled [89]. Following the permission by the USFDA, food products from the US may contain up to 1% of TiO_2_ nanoparticles [78], while previously, the European Union (EU) allowed the use of TiO_2_ at quantum satis [90]. By October 2021, the EU Commission—based on a new evaluation of risks of TiO_2_ by the EFSA—banned TiO_2_ from being used as a food additive from 2022.

Dietary TiO_2_ intake seems to be culturally different, and consumption ranges from micrograms to milligrams per kilogram body weight (kg_BW_), depending on nutritional and personal habits [75,76,81,91]. Germany’s mean dietary TiO_2_ intake ranges between 0.5 and 1 mg TiO_2_/kg_BW_ for adults and 2 mg TiO_2_/kg_BW_ for children [91]. For the US population, the daily TiO_2_ exposure is believed to range between 1 and 2 mg TiO_2_/kg_BW_ for children and 0.2 and 0.7 mg TiO_2_/kg_BW_ for adults [92]. With children consuming 2–3 mg TiO_2_/kg_BW_/day and adults ingesting 1 mg TiO_2_/kg_BW_/day, TiO_2_ consumption is even higher in the United Kingdom [92]. High TiO_2_ contents were evidenced in chewing gums, candy, and fine bakery wares [75], reaching up to 2.5 mg TiO_2_ per gram of food [75,91]. Due to their preference for sweets, children and teenagers are highly exposed. Their estimated maximum consumption is up to 32.4 mg TiO_2_/kg_BW_/day [75,76]. Chewing one single bubblegum might result in an intake of 5 mg TiO_2_, while powdered donuts can contain up to 100 mg TiO_2_ per serving [92].

A risk assessment conducted by the FDA in 1969 classified TiO_2_ to be safe in use, and the definition of an acceptable daily intake was considered unnecessary [93]. However, this decision was based on only five publications, reporting low solubility of the compound and absence of significant effects in animal experiments, suggesting low bioactivity, absorption rates, and minor accumulation in the body [77]. Re-evaluations by the Scientific Committee on Food (SCF) in 1975 and 1977 did not entail any regulatory limits for the use of TiO_2_ [90]. In 2010, the International Agency for Research on Cancer (IRAC) classified TiO_2_ as a human carcinogen in response to sufficient evidence that inhalation of TiO_2_ nanoparticles promotes lung cancer [94]. In recent decades, many experts in the field expressed their concerns about the safety of TiO_2_, initiating a re-evaluation of the use of TiO_2_ nanoparticles as a food additive by the EFSA in 2016. Although the available data were considered insufficient and not of concern, a safety margin of 2.25 mg TiO_2_/kg_BW_/day was introduced [90]. In 2018, the discussion was re-opened when the EFSA followed a request from the European Commission to deliver a scientific opinion regarding the safety of TiO_2_ in food products. With only four publications considered relevant [95,96,97,98], the committee concluded that a re-evaluation of the current opinion was not required considering the outcome of the studies [90]. Other valuable studies that indicate severe impacts on human health by consuming TiO_2_ nanoparticles were neglected [77,92,99,100,101,102].

The discussion was recently re-opened, and voices were raised demanding the ban of TiO_2_ from food. On the first of January 2021, the French government followed a recommendation from the French Agency for Food, Environmental and Occupational Health and Safety (ANSES) to prohibit the sale of all food products containing TiO_2_ [103,104]. In an open letter to the European Commission, more than 26 European and national non-governmental organizations called for a general Europe-wide ban of TiO_2_ in food [105]. By the end of 2020, the European Parliament called on the European Commission to apply the “precautionary principle” and to remove TiO_2_ from the EU list of permitted food additives [106,107]. A recent reassessment of TiO_2_ on the part of the EFSA raised severe doubts about the safety of TiO_2_ as a food additive and consequently resulted in a ban of TiO_2_ from food products within the EU earlier this year.

## 4. TiO_2_ Effects on Gut Homeostasis—New Insights

TiO_2_ nanoparticles are heavily used in comestible goods, and exposure can occur via oral uptake, skin contact, or inhalation. Due to their small size, TiO_2_ nanoparticles penetrate the gastrointestinal barrier [108,109]. For the broad public, the relevant route of TiO_2_ uptake is ingestion of nanoparticles with the food. TiO_2_ nanoparticles pass through the GIT following oral uptake, where the food matrix and biopolymers can alter their physicochemical properties, influencing their gastrointestinal fate [110,111].

**Figure 2:**
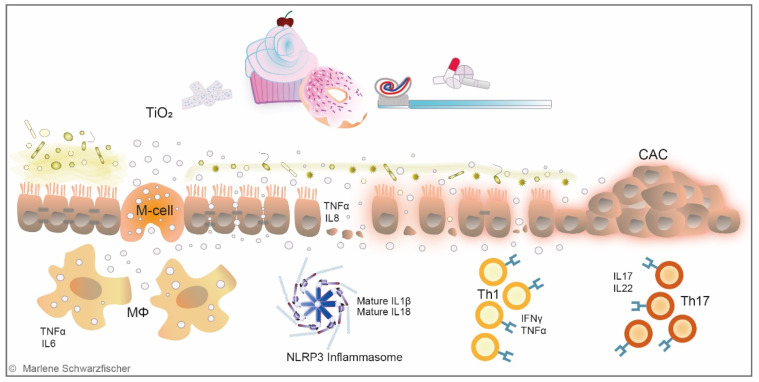
TiO_2_—impact on gut homeostasis. Food-grade titanium dioxide (TiO_2_) nanoparticles are omnipresent in the Western diet. Ingested TiO_2_ nanoparticles can enter the system through microfold cells (M-cells), paracellular transport through epithelial cells or penetration through epithelial lesions. In the gut, TiO_2_ exposure negatively affects microbiota diversity/composition and has adverse impacts on barrier integrity via reduction in mucus secretion, modulation of tight junctions, and induction of pro-inflammatory cytokine expression in enterocytes. TiO_2_ nanoparticles accumulate in phagocytic macrophages (MФ), promoting pro-inflammatory response. Furthermore, TiO_2_ nanoparticles activate the NLRP3 inflammasome in epithelial and immune cells, triggering pro-inflammatory signaling cascades. TiO_2_ exposure induces clonal expansion of T-helper (Th1/Th17) cells in the lamina propria, which exert pro-inflammatory functions and are closely associated with IBD pathogenesis. Together, these events promote chronic epithelial lesions and intestinal inflammation, resulting in an elevated risk of developing colitis-associated colon carcinoma (CAC).

Currently available data from animal models indicate that the majority of ingested TiO_2_ nanoparticles is not absorbed into the system but is excreted with the feces [112,113,114,115,116]. A human volunteer study testing different particle sizes (15 nm, 100 nm, 5000 nm) did not detect increased TiO_2_ levels in the serum after single oral TiO_2_ exposure, independent of the particle size [113]. Strikingly, however, TiO_2_ nanoparticles were evidenced in human livers and spleens, indicating that they accumulate in the human body [117]. In mouse models, TiO_2_ accumulation was found in the GIT, brain, blood, liver, spleen, kidneys, heart, and lung tissues after oral longer-term exposure [96,108,109,118,119,120,121,122,123]. Three pathways for particle translocation from the GIT into the system have been identified so far: paracellular transport across tight junctions, transcytosis across M-cells in Peyer’s patches, and persorption across degrading enterocytes [108,124] (Figure 2).

Following multiple approaches, Brun et al. showed that TiO_2_ nanoparticles cross the ileum epithelium and Peyer’s patches, inducing epithelial impairment and chronic damage [108] (Figure 2). These findings are supported by in vitro studies, showing that TiO_2_ nanoparticles disrupt tight junctions between intestinal epithelial cells and induce the expression of pro-inflammatory cytokines [125]. Furthermore, it was demonstrated that TiO_2_ application in vitro and in vivo significantly decreased mucus secretion [126,127] (Figure 2). Interestingly, intestinal biopsies sampled from IBD patients revealed aggregates of TiO_2_ nanoparticles in M-cells and underlying macrophages of gut-associated lymphoid tissue [128], where the earliest signs of lesions in CD are usually observed [119,129]. Follow-up studies reported a disruption of systemic or gastrointestinal immune homeostasis and microbiota dysbiosis upon TiO_2_ administration [96,130,131,132]. Li et al. were the first to associate TiO_2_ with the gut microbiome [131]. In line with these findings, Mu et al. and Pinget et al. reported microbiota dysbiosis and altered release of bacterial metabolites following TiO_2_ intake [130,132] (Figure 2). Nogueria et al. were the first to describe Th1-mediated inflammatory response in the small intestine of mice orally treated with TiO_2_ nanoparticles [133]. Bettini et al. observed an exceeding Th1/Th17 immune response in the Peyer’s patches from rats orally exposed to TiO_2_, resembling Th17-driven autoimmune diseases in humans [96] (Figure 2). Talamini et al. reported intestinal inflammation upon TiO_2_ exposure [120,132]. Recent studies revealed dysfunction of the epithelial barrier in the ileum, probably induced by a Th1/Th2 imbalance and/or increased lipopolysaccharide (LPS) signaling upon TiO_2_ administration [134,135]. Studies performed by Huang et al. indicate that TiO_2_ nanoparticles prime an abnormal activation state of macrophages, characterized by an excessive pro-inflammatory phenotype and suppressed innate immune function [136]. Furthermore, TiO_2_ nanoparticles were shown to cause mitochondrial dysfunction, induce oxidative stress and attenuate phagocytotic capacities of macrophages [136]. In 2017, our group detected increased TiO_2_ serum levels in UC patients undergoing an acute phase of the disease. Oral administration of TiO_2_ nanoparticles in a mouse model of acute DSS-induced colitis triggered assembly of the NLR pyrin domain-containing 3 (NLRP3) inflammasome and release of the pro-inflammatory cytokines interleukin (IL)-1β and IL-18 resulted in aggravation of intestinal inflammation [119]. In line with these findings, Mu et al. described an aggravation of chronic DSS colitis and reduced CD4+ T-cells and Tregs populations in mesenteric lymph nodes (MLN) [130]. Contrary, in a very recent study from Gao et al. TiO_2_ administration ameliorated Trinitrobenzenesulfonic acid solution (TNBS)-induced colitis. The authors describe that TiO_2_ nanoparticles decreased TNF expression in the colon and beneficially modified the microbiome, including depletion of pathogenic commensal strains [137].

In addition to their IBD promoting potential, food-grade TiO_2_ nanoparticles were shown to exacerbate tumor formation in the DSS azoxymethane (AOM) [138] and the dimethylhydrazine (DHM) colon cancer model [96]. IBD with colonic involvement predisposes patients to develop colitis-associated cancer (CAC) due to the chronic inflammatory state [139,140,141,142]. It has been reported that CD increases the risk of developing colorectal cancer (CRC) by roughly 1.8 times and UC even up to 8 times compared to the risk of developing CRC in the general population [143]. CAC represents about 2% of all CRC cases [144], and it has been extensively reported that patients with IBD who develop CAC are frequently diagnosed at an advanced stage and face a worse prognosis than those with CRC without IBD [145,146,147,148].

Ingestion of TiO_2_ nanoparticles causes impairment of innate and adaptive immune system, microbial dysbiosis, and breakdown of the intestinal epithelial barrier—characteristic key events of IBD pathogenesis. Chronic dietary exposure with TiO_2_ nanoparticles may disrupt intestinal homeostasis and low-grade inflammation in healthy individuals. In IBD patients, translocation of TiO_2_ nanoparticles might be substantially increased due to disruption of the intestinal barrier, causing an amplification of these processes and exacerbation of inflammation. Therefore, food-grade TiO_2_ nanoparticles might be an underrated IBD trigger and a potential risk for healthy individuals and patients suffering from intestinal diseases. Since TiO_2_ nanoparticles in food and comestible goods only serve the purpose of increasing the appeal and the commercial value of certain products, this health risk seems unnecessary and unreasonable. By the first of January in 2022, TiO_2_ was banned as a food additive by the European parliament. However, it is still present in cosmetic products, such as toothpaste, or pharmacological preparations, such as tablets or pills.

## 5. Global Plastic Crisis—Intestinal Consequences?

Apart from food additives, plastic particles are also consumed unknowingly with our diet. After decades of environmental pollution with plastic products of all kinds and shapes, concerns are rising that this global load of plastic might impact marine and terrestrial life. Nonetheless, production volume and consumption are continuously expanding [149], and the accumulation of plastic debris is increasing all over the planet [150,151]. The most commonly used plastic polymers are low-density polyethylene (LDPE), high-density polyethylene (HDPE), polypropylene (PP), polystyrene (PS), polyethylene terephthalate (PET), and polyvinyl chloride (PVC). In contrast, other polymers, such as nylon, acrylic, polyurethanes, polylactic acid, polycarbonate, and other plastics are also present [152]. By 2019, 368 million tons of plastic had been produced [149], of which approximately more than 8 million metric tons are entering the oceans every year [151].

**Figure 3:**
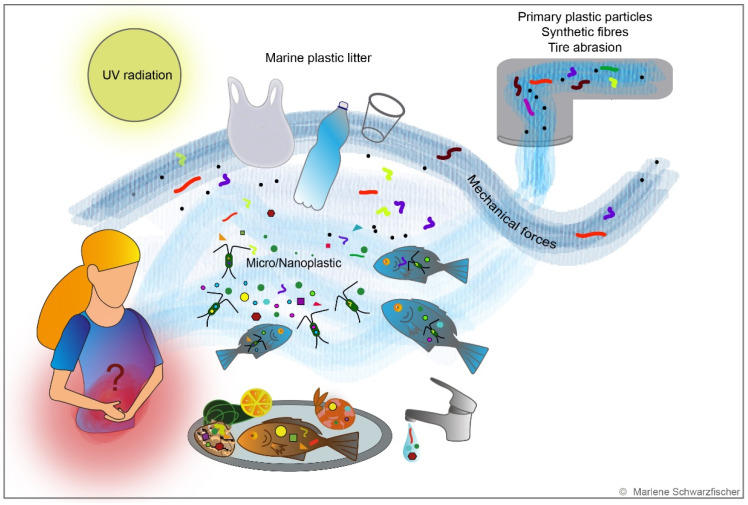
Nano- and microplastics in the environment. Several sources contribute to plastic contamination of fresh and salt water. Primary MP particles are designed for commercial use and encountered in cosmetic and pharmaceutical products. In contrast, secondary MP plastic particles progressively emerge with plastic fragmentation under the influence of environmental conditions. UV radiation, mechanical forces, biological degradation, and embrittlement result in the formation of NP and MP particles, which are ingested by marine zooplankton and consequently enter the marine and human food chain. Microplastic contamination was evidenced in seafood, drinking water, and other foodstuff. However, the impacts of ingested plastic particles on human health are not yet understood.

Attention is mainly directed on small plastic particles. Primary plastic particles are introduced to fresh and saltwater as manufactured micro-scale polymers in cosmetic care products or medicine [153,154,155]. In contrast, secondary particles emerge due to progressing fragmentation of plastic litter [156,157] (Figure 3). A substantial part of secondary plastic particles derives from public trash, inadequate waste disposal by the fishing industry [158], and sewage water containing plastic particles generated from washing synthetic textiles [159] or tire abrasion [160] (Figure 3). Environmental impacts, including UV radiation, mechanical forces, biological degradation, and embrittlement, result in the formation of so-called microplastic (MP) particles with diameters < 5 mm [161,162]. These microplastic particles can be further fragmented into nanoplastic (NP) particles (Figure 3). The exact definition of NP is currently controversially discussed. MP particles have been discovered all over the globe, including beaches, shores, fresh and salt water, deep-sea sediments, and even in the Antarctic sea ice [154]. The load of MP debris in the oceans may differ geographically [152].

The effects of NP and MP exposure on aquatic organisms have been intensively studied. MP particles were shown to cause oxidative stress, epithelial damage, intestinal inflammation, and consecutive mortality in zebrafish *Danio rerio* [163,164]. Furthermore, MP exposure impacts the energy homeostasis of marine species by reducing energy intake due to decreased feeding activity, reduced predatory performance, and adverse effects on digestion, such as inhibition of digestive enzymes and therefore impaired uptake of nutrients [165,166,167,168,169,170]. Moreover, MP ingestion resulted in a negative energy balance due to increased energy consumption, resulting from inflammatory response, increased gut residence time and excretion mechanisms [165,166,168,169], and metabolic changes [170,171,172]. In mussels, MP exposure resulted in immunosuppression and tissue-dependent modulation of the immune response [173,174].

NP and MP exposure occurs via inhalation, ingestion, or dermal contact. Washing and shedding synthetic textiles abrasion of tires and commodities generates air-born NP and MP particles, small enough to enter the respiratory tract [166,175]. Prata et al. estimated that human beings inhale 26–130 MP particles per day [176], while studies by Vianello et al. suggested an average intake of 272 MP particles a day [177]. Respiratory uptake of MP particles might be even higher in individuals working in synthetic textile, flocks, and the vinyl chloride or polyvinyl chloride industries, where respiratory diseases among the workers are frequently described [178,179]. In 1998, Pauly et al. evidenced plastic fibers in human lung and lung cancer biopsies [180]. In rat models, intratracheal exposure resulted in biochemical and histopathological changes of rat lungs due to acute inflammatory response [181,182].

Ingestion is considered the primary route of human plastic exposure [183]. It is estimated that, on average, an adult person consumes around 39,000–52,000 particles a year [184] or 5 g of plastic every week [185]—the equivalent of a credit card. In addition, MP has been evidenced in human feces [186] and all human organs [187]. Although most studies investigating the effect of plastic particles on the environment have been conducted in the marine ecosystem, plastic contamination might be more pervasive in the terrestrial ecosystem and human diet than expected. Small plastic particles are ingested by aquatic organisms, enter the circulatory system of marine wildlife [163,188,189,190,191,192], and then spread throughout the marine food chain, contaminating seafood which ends up on the markets for human consumption [193,194,195] (Figure 3). Although plastic packaging for drinks and food is an obvious source of ingested NP and MP particles [196,197,198,199], MP particles have also been found in tap water, honey, sugar, sea salt, and beer [200,201,202,203,204].

So far, the absorption and translocation of NP and MP particles in the human body have not been thoroughly investigated. However, it is likely that due to their similar size and chemical inertness, plastic particles take the same routes as TiO_2_ nanoparticles [124]. Plastic research is still in its infancy, and the impact of NP and MP exposure on human health is hardly predictable. The toxicity of plastic particles might depend on their chemical composition and is assumed to have a dose-dependent, accumulative effect [73,74]. In vitro studies demonstrated that human immune cells and intestinal and lung epithelial cells internalize MP particles, which results in oxidative stress, endoplasmatic reticulum (ER) stress, and autophagic cell death [32,205,206]. Plastic was shown to accumulate in the liver, kidney, and gut of mice fed with MP particles [29,171]. Several studies describe adverse health effects in rodents orally exposed to MP particles.

Very little data are available on NP particles, although they may possess increased bioactivity due to their small size. Deng et al. were the first to describe the accumulation of MP particles in the liver, kidney, and the GIT and its impacts on energy and lipid metabolism, oxidative stress, and neurotoxicity caused by PS MP particles [171]. However, this study was critically discussed [207,208]. A follow-up study described adverse impacts of PS particles on the reproductive system of male mice caused by oxidative stress and activation of mitogen-activated protein kinase (MAPK) P38 [209]. This study was supported by Hou et al., Park et al., and Haibo et al. describing reproductive and developmental toxicity of PS [210,211] and PE particles [210]. Furthermore, Hou et al. reported adverse effects of PS MP on the ovary of mice, potentially resulting in female infertility [212]. Luo et al. exposed pregnant mice to PS MP particles and observed metabolic disorders in their offspring, indicating that MP particles might cross the placenta [213]. Luo et al. reported altered metabolic homeostasis in the offspring of PS MP exposed mice [214]. In a physiological approach by Preia de Costa Araújo and Malafaia, tadpoles were exposed to MP particles, fed to fish, and plastic-contaminated fish were fed to experimental mice. MP was found to accumulate in tadpoles, fish, and mice, proving translocation of MP from the aquatic to the terrestrial food chain. Furthermore, MP was shown to induce behavioral disorders in mice [215]. Overall, the impact of MP particles on rodents was relatively mild compared to the effects of MP exposure in aquatic species. Discrepancies between rodents and fish might result from anatomical differences, whereby fish gills allow multiple routes for plastic uptake and accumulation. In contrast, plastic uptake in rodents is limited to the gastrointestinal route [216].

**Figure 4:**
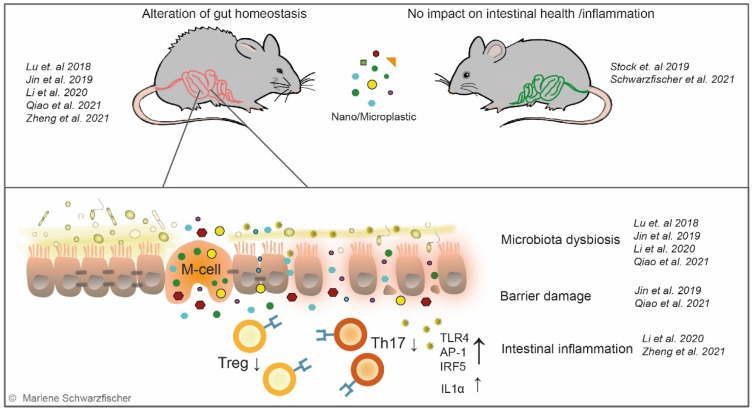
Nano- and microplastics—impact on gut homeostasis. The impact of nano- and micro-sized plastic particles on the intestine is controversially discussed, and the number of studies investigating these relations is limited. It is assumed that small plastic particles take the same routes as TiO_2_ nanoparticles and penetrate the intestinal epithelium. Although two publications did not detect any effects of NP or MP on intestinal health and/or inflammation, respectively, conflicting studies report alteration of the gut homeostasis upon plastic administration in mouse models. Several studies describe adverse effects on the gut microbiome, reduced mucus secretion, and damage of barrier integrity upon administration of MP particles. Li et al. describe the reduction in regulatory T-cells (Treg) and Th17 cells, increased Toll-like receptor 4 (TLR4) downstream signaling, and IL1α expression, suggesting activation of pro-inflammatory cascades and translocation of bacterial products into the system [28,29,30,31,32,217,218].

The impact of small plastic particles on intestinal health is controversially discussed and different in vivo mouse experiments obtained contradictory results. So far, only a few studies—using different types and sizes of plastic and various exposure times—investigating the impact of plastic particles on the gut have been conducted (Table 1). Lu et al. reported that oral plastic exposure induced hepatic lipid disorder in mice. Furthermore, they detected alterations in mucus secretion and changes in the richness and diversity of the gut microbiota characterized by a decrease in relative abundances of Firmicutes and α-Proteobacteria [28] (Figure 4). Jin et al. described the breakdown of the epithelial barrier, gut microbiota dysbiosis, and impacts on the metabolic pathways in the microbial community in mice treated with MP particles [29] (Figure 4). At the same time, Li et al. [30] and Qiao et al. [217] characterized similar effects upon treatment with MP particles (Figure 4). Strikingly, Zheng et al. reported that MP administration aggravated acute DSS colitis and increased intestinal epithelial permeability [31]. Controversially, Stock et al. did not detect histological changes or inflammatory response in the intestine after exposure to MP particles [32] (Figure 4). Within our work we investigated the effect of plastic nanoparticles on intestinal health and evaluated their inflammatory potential in acute and chronic models of colitis. Surprisingly, in vivo experiments performed by our group did not reveal adverse effects on gastrointestinal health nor gut inflammation. Although, we evidenced NP and MP the system, long-term administration of nano- or micro-sized PS particles did not alter gut homeostasis, nor did it promote acute or chronic DSS colitis [218] (Figure 4). Nevertheless, our data align with previous studies, reporting an accumulation of ingested plastic particles in organs distant from the gut [29,175]. Limiting our study to a colonic IBD model, we cannot exclude the potential effects of plastic ingestion on the small intestine, primarily affected in CD [219].

Consumption of small plastic particles is rising with the increasing abundance of plastic in the human diet and toxicity might be increasing with an accumulation of plastic particles in the body. Therefore, studying the effects of plastic exposure on human health is of great interest. IBD patients might be particularly vulnerable to the adverse effects of ingested plastic particles as disruption, and increased permeability of the intestinal epithelial barrier [220] might enable translocation of NP and MP particles to a greater extent. However, a potential disease-promoting effect of plastic particles in IBD requires further investigation.

## 6. Summary

The presence of nano-and microparticles in the human diet has recently gained public awareness and raised significant concerns. Their impact on human health is not yet fully understood and controversially discussed. Although TiO_2_ nanoparticles, designated as food-coloring agent E171, are systematically applied to food products for cosmetic reasons, plastic particles in drinking water and food stuff are the consequence of longstanding environmental pollution. Breakdown of the intestinal epithelial barrier and aberrant immune responses are key events in the pathogenesis of IBD. Epithelial lesions might enable translocation of particles into the system, where they might trigger an excessive immune response. Therefore, IBD patients might be particularly vulnerable to adverse health effects caused by the ingestion of nano- and microparticles.

Just recently, the European Union took action and banned TiO_2_ nanoparticles from food production—a decision which was built upon a body of literature, raising serious doubts about the safety of food-grade TiO_2_. With respect to intestinal diseases, several good studies demonstrated the inflammatory potential of TiO_2_ nanoparticles and reported negative impacts on the intestinal barrier and the gut microbiome. Although the scientific evidence on the harmful properties of TiO_2_ nanoparticles seems to be explicit, the process of understanding the effects of dietary nano- and microplastic particles on the human body is still at the very beginning. The impacts of plastic particles on epithelial integrity, gut homeostasis, and intestinal inflammation are controversially discussed and require further investigation.

## Figures and Tables

**Table 1:** Literature Search: Impact of NP and MP on intestinal health of rodents.

Publication	Type	Size	Dose	Administration	
Lu 2018 [28]	PS	0.5 μm and 50 μm	100, 1000 μg/L	Drinking water	Continuous 5 w
Jin 2019 [29]	PS	5 μm	1000 ug/L	Drinking water	Continuous 6 w
Stock 2019 [32]	PS	Mixture: 1, 4, 10 μm	10 mL/kg_BW_	Gavage	3× per week 4 w
Li 2020 [30]	PE	Mixture: 10–150 μm	6, 60, 600 μg/day	Drinking water	Continuous 5 w
Zheng 2021 [31]	PS	5 μm	500 ug/L	Drinking water	Continuous 4 w
Qiao 2021 [217]	PS	70 nm and 5 μm	0.2 or 2 mg/kg_BW_	Gavage	1× per day 4 w
Schwarzfischer 2021 [218]	PS	50 nm and 1 µm	0.2 mg/day	Drinking water	Continuous 24 w
